# Fat Addiction: Psychological and Physiological Trajectory

**DOI:** 10.3390/nu11112785

**Published:** 2019-11-15

**Authors:** Siddharth Sarkar, Kanwal Preet Kochhar, Naim Akhtar Khan

**Affiliations:** 1 Department of Psychiatry and National Drug Dependence Treatment Centre (NDDTC), All India Institute of Medical Sciences (AIIMS), New Delhi 110029, India; sidsarkar22@gmail.com; 2 Department of Physiology, All India Institute of Medical Sciences (AIIMS), New Delhi 110029, India; kpkochhar6@gmail.com; 3 Nutritional Physiology and Toxicology (NUTox), UMR INSERM U1231, University of Bourgogne and Franche-Comte (UBFC), 6 boulevard Gabriel, 21000 Dijon, France

**Keywords:** diet, fat, food addiction, obesity

## Abstract

Obesity has become a major public health concern worldwide due to its high social and economic burden, caused by its related comorbidities, impacting physical and mental health. Dietary fat is an important source of energy along with its rewarding and reinforcing properties. The nutritional recommendations for dietary fat vary from one country to another; however, the dietary reference intake (DRI) recommends not consuming more than 35% of total calories as fat. Food rich in fat is hyperpalatable, and is liable to be consumed in excess amounts. Food addiction as a concept has gained traction in recent years, as some aspects of addiction have been demonstrated for certain varieties of food. Fat addiction can be a diagnosable condition, which has similarities with the construct of addictive disorders, and is distinct from eating disorders or normal eating behaviors. Psychological vulnerabilities like attentional biases have been identified in individuals described to be having such addiction. Animal models have provided an opportunity to explore this concept in an experimental setting. This discussion sheds light on fat addiction, and explores its physiological and psychological implications. The discussion attempts to collate the emerging literature on addiction to fat rich diets as a prominent subset of food addiction. It aims at addressing the clinical relevance at the community level, the psychological correlates of such fat addiction, and the current physiological research directions.

## 1. Introduction

Over the last half a century, many developing countries have seen rapid socio-economic development, resulting in a move from a traditional to a modern way of life, including changes in local dietary and culinary profiles [1,2,3]. Abundance and easy availability of food, especially the one that is rich in fat and carbohydrate, have resulted in changes of dietary patterns and preferences. Right from early childhood in developing brains, the exposure and imprinting to high sugar, high salt and high fat food (rich in saturated and trans-fat), which is cheap and easily available, are impacting the health of younger population. Trans fat may lead to its greater consumption than polyunsaturated fat, as the latter is more quicker than the former to trigger satiety [4]. The changes in dietary intake profile with cultural and societal transitions have gained traction [5]. The dietary profile and constitution have a role in the etiopathogenesis of lifestyle-related diseases like obesity, metabolic syndrome, coronary artery disease, gut motility disorders, psychosomatic, autoimmune as well as degenerative disorders. Major transition, noticed during the last couple of years, has been an increasing use of sugar, processed food, beverages, animal-fat based food rich in trans-fats that have impacted human health [6,7,8,9,10].

In the recent years, there is a growing interest in the concept of food addiction from both clinical and applied nutritional research perspectives [9,11,12,13,14]. The increase in obesity, and associated metabolic syndrome and diabetes mellitus have called into the questions about factors leading to genesis of obesity. The imbalance of energy intake has been proposed as one of the reasons of increasing prevalence of obesity, though there are other several factors, i.e., epigenetics, psychological trauma, use of medications and dieting, that may increase body weight gain. why do some individuals consume excess of certain types of food (including fat-rich food)? Hence, the phenomenon of addiction to food has been suggested to be one of the mechanisms. Food addiction can refer to a variety of substrates, but fat and sugars have been considered the typical prototype food items to which individuals develop addiction. The occurrence of distinct features of salience and inability to control intake of specific types of foods have been considered similar to addiction to other psychoactive substances. The adverse consequences of uncontrolled fat intake on the body metabolism have been documented [15,16]. This has implications in intervention modules for addressing this problem and promoting healthy lifestyles [17,18]. Yet, the understanding of fat addiction as a concept is still under evolution, and progress is being made to characterise and discern the psychology and physiology behind this condition.

Recent studies suggest that fat has its own metabolic, physiological and nutritional profiles, which are distinct from other macronutrients [19,20,21]. Fat accords palatability and organoleptic properties to food, and is consumed across all ages from infancy through adulthood to elderly. Evolutionarily, this confers survival benefit due to high energy density, more so in cultures with thrifty genotypes [22,23,24]. In recent years, the benefits of mono-unsaturated fatty acid and controlled amounts of saturated fat intake have been revisited, especially in the context of benefits in cognitive functioning, synaptic connectivity, and membrane stability for both brain and heart health [25,26].

In this context, taste for fat has been proposed as the sixth taste modality in recent years [27]. The interactions between fatty acids and specific receptors in taste bud cells elicit physiological changes that are implicated in dietary fat preference via the activation of tongue-brain-gut axis. This phenomenon has an implication in the genesis of obesity as oro-sensory detection of nutrients determines the ‘liking’ and ‘wanting’ of food products. It has been proposed that there are two components of eating behavior, represented in neuronal circuits, i.e., emotional (hedonic and affective) and metabolic (homeostatic). Obesity may arise due to the imbalance of these two eating motives.

Research on food addiction or eating addiction has not paid distinct attention to specific nutrients like dietary fat [9,10,12,13]. The concept of fat addiction would have important psychological determinants like motivation, depression, anxiety and reasoning that merit cautious evaluation. There is cognitive appraisal that makes an individual “like” and “want” a specific food product, and the reward obtained from the food is cognitively processed as well. Hence, the intertwined psychological and physiological aspects of addiction towards fat rich food must be considered and understood further. There is a lack of comprehensive synthesis of literature to provide an account of fatty food addiction. In this paper, we have aimed at providing an overview of the construct, the clinical relevance, the psychological correlates, and the current physiological research trajectory in the emerging area of fat addiction as a subset of food addiction. Wherever specific literature with regard to fat is not available, evidence related to food addiction will be alluded to. However, our main emphasis is to discuss about fat addiction as this phenomenon might lead to high dietary fat intake and, consequently, to obesity. The term “high fat” in this article would mean the diet where the calories brought by fat are more than 40% of total dietary calories as most of industrialized countries recommend to respect this limit. As we have mentioned in the title, our main emphasis is to shed light on fat addiction and we have excluded other addictive behaviors like sweet addiction.

## 2. The Construct of Fat Rich Food Addiction

Addressing the issue of obesity would require improved knowledge of pathophysiological and neurobehavioral mechanisms. This would help better target behaviors which predispose individuals to obesity [28]. Schmidt and Campbell argue that disordered eating cannot remain “brainless” [29], and the “psychological constructs”, that define aberrant consumptive patterns of food, are relevant. In this regard, addiction to food explains hedonistic excess and uncontrolled consumption of food items which are associated with adverse consequences.

Addiction towards fat rich diet relates to the overall definition of addiction. Addiction has been conceptualized as a maladaptive pattern of substance intake or behavior that signifies neurobiological changes and is associated with adverse consequences. The nosological systems providing nomenclature to diagnosis has moved on from abuse and dependence to substance use disorders in DSM5. The criteria based evaluation of the cluster of symptomatology helps provide with coherent account of the disorder, and categorize individuals who meet a threshold for diagnosis and consequent potential treatment.

### 2.1. Defining Fatty Food Addiction in the Context of Nutrient Intake

Food addiction shares some of the commonalities with drug addiction like craving, bingeing and tolerance [30]. The DSM5 criteria for substance use disorders have been adapted and explored in the context of food addiction [31,32]. The 11 criteria for substance use disorders can be applicable to individuals with addiction to lipid dense foods (especially trans and saturated fat). The empirically supported criteria describe a substance (food) often taken in larger amounts or over a longer period of time than that was intended, persistent desire or unsuccessful efforts to cut down or control substance use (food), and continued use despite knowledge of having a persistent or recurrent physical or psychological problem. The plausible features include great deal of time being spent in activities necessary to obtain or use the substance (food) or recover from its effects, recurrent substance (food) use resulting in a failure to fulfil major role obligations, continued use despite having persistent or recurrent social or interpersonal problems, important social, occupational, or recreational activities are given up or reduced, and tolerance. What might be difficult to clearly clinically elicit are withdrawal (while differentiating from energy deficit), and recurrent use in physically hazardous situations. As with different substances, each of the criteria is endorsed to different extent by a sample of participants.

The diagnostic constructs related to food addiction include binge eating disorder and an eating disorder not otherwise specified. Binge eating disorder is characterised by repeated ingestion of eating in large amounts of food in a short amount of time, followed by intense guilt and attempts to either remove the food (by vomiting or using laxatives) or compensatory behaviors to increase the energy expenditure [33,34]. On the other hand, eating disorder not otherwise specified is a diagnostic rubric that resembles anorexia nervosa or binge eating disorder, but does not fulfil the diagnostic thresholds for these disorders. These disorders may have some overlap with food addiction from a phenomenological and behavioral perspective, but the constructs themselves are distinct. It has been seen that individuals with binge eating disorders have greater rates of food addiction, than expected by chance [35,36], though at the same time, not all individuals with binge eating disorders would have food addiction [36,37].The main point of divergence lies in the focus of the constructs: food addiction lays emphasis on the salience and loss of control of hedonic eating behaviors, while eating disorders are accompanied by intense immediate guilt after excessive food consumption and efforts are made to get rid of (effects of) the ingested food quickly.

### 2.2. Clinical and Epidemiological Implications of Addiction towards Fat

While limited literature has looked at addiction to fat rich foods per se, there is enough evidence that has ascertained the occurrence rate and determinants of food addiction in the community and clinical samples [38,39]. The questionnaires used to assess food addiction generally incorporate fat as a component of food that the respondents are asked to think about, when they answer the questions. The Yale Food Addiction Scale is perhaps the most commonly used instrument for the assessment of food addiction. The weighted mean prevalence of food addiction according to this instrument was 19.9% [38]. The prevalence of food addiction was high in women with obesity [38]. Also, food addiction was higher in clinical samples, as compared to community samples [37,38]. Food addiction was high in subjects that were either obese, or suffered from eating disorders. High scores of food addiction were associated with high depressive symptoms, food craving and impulsivity. Food addiction has not only been related to negative mood states, but also with poorer quality of life [40]. It has been seen that individuals with food addiction had higher dietary fat intake as compared to those without food addiction [41]. Similarly, Pursey et al. reported that the subjects with high food addiction scores had high percentage of consumption of saturated fat [42]. Thus, food addiction provides a paradigm for identification of individuals with skewed dietary profiles with other psychological vulnerabilities, which might require concomitant attention.

Food addiction has also been studied in those individuals who have undergone bariatric surgery which is generally indicated for people with severe obesity [43]. The rates of food addiction in bariatric surgery population go down after the surgery. In one study, the proportion of individuals with food addiction reduced to 2% post-surgery from 32% pre-surgery [44]. Another long term follow-up suggested that the rates of food addiction reduced from 57.8% to 7.2% at 6 months and to 13.7% at 12 months after surgery [45]. In pre-operative cases of bariatric surgery, the dietary intervention is less effective in individuals with food addiction [46]. It has been seen that food addiction in bariatric surgery patients was associated with greater levels of depression, anxiety and binge eating episodes, though it did not predict the degree of weight loss. Thus, it seems that food addiction has some clinical prognostic influence with surgical intervention outcomes.

### 2.3. Measurement Approaches

Currently the standard of practice for determination of food addiction has been the diagnostic cut-off from the Yale Food Addiction Scale (YFAS) [47]. The YEAS is a 25 item self–reported questionnaire based scale that assesses various features of food addiction. There are two items that assess for clinically significant impairment or distress. The instrument looks at the past year pattern of food intake and includes fatty foods like steak, bacon, hamburgers, cheeseburgers, pizza, and French fries as one of the representative group of foods that are mentioned in the questionnaire. The instrument has become standard of use in the field of food addiction. The instrument has adequate internal reliability, good convergent validity and good discriminant validity. The instrument has been adapted for use in children [48]. The instrument has also been translated into several other languages like Chinese, French and Malay [49,50,51]. A newer version of the scale (YFAS 2.0) has been developed considering the changes in conjunction with the DSM5 [52]. The instrument has been used in studies of epidemiology, etiology, nosology and interventions of food addiction. While the YFAS addressed food as a whole, assessing fat addiction separately may have implications for interventions. This could be in terms of the type of food products that are focused upon in the intervention modules that are developed. This would have also a corollary for the investigation procedures that can include assessment of salience and behavioral neuroplasticity (eye tracking and neuroimaging) for different types of food products (fat rich versus carbohydrate rich, sweet versus savory fatty food) that are implicated in food addiction.

Other self-reported scales and questionnaires for assessment of aspects of food addiction are also available and have been validated, though they rely on features like craving and eating patterns. These include Eating Behaviors Questionnaire [53], Food Cravings Questionnaire [54], Eating Behaviors Patterns Questionnaire [55], and Power of Food Scale [56]. Many of these questionnaires are self-reported, i.e., the individual reads through the questions and responds through them. The responses are thereafter graded and interpreted based upon the cut-offs from the population scores.

## 3. Psychological Correlates of Addiction to Fat Rich Diets

### 3.1. Attentional Biases and Cognitive Functioning

Research has been carried out towards attentional biases and psychological processing in individuals with food addiction. Obese as compared to lean teens showed less activation of prefrontal regions (dorsolateral prefrontal cortex, ventral lateral prefrontal cortex) when trying to inhibit responses to high-calorie food images which suggest behavioral evidence of reduced inhibitory control [57]. Adults who had greater dorsolateral prefrontal cortex activation when instructed to “resist craving” after viewing food images had better weight loss success following gastric bypass surgery [58]. This suggests that visual cue induction paradigms have relevance to assessment of how food images are processed centrally.

Rodrigue et al. [59] compared those with higher and lower food addiction scores on cognitive processes of planning, inhibition, cognitive flexibility and error processing. The investigators found that high food addiction group differed from the low food addiction group only in terms of inhibition/cognitive flexibility scaled scores, but not in individual scores. The authors infer that though basic level processes are intact, individuals with higher food addiction scores experience greater difficulties in more challenging context where they had to simultaneously keep in mind to inhibit a behavior and switch their mind-set when the task required it. This might make it difficult for them to anticipate the long-term consequences of behavior. Also, individuals with symptoms of food addiction made more errors as the interference task became challenging, suggesting that those with food addiction might have greater difficulty in detecting and monitoring errors. Another study compared error monitoring among individuals with food addiction and healthy controls using the Eriksen flanker task [60]. The results suggested that food addiction group had higher number of errors on the flanker task, implying impaired performance monitoring and cognitive control, as seen with other addictions. In a study that included women with obesity, food addiction severity levels were negatively correlated with overall scores on the Iowa Gambling Task, which measures decision making capacity [61]. Also, those with food addiction had attentional deficits as reflected by more omissions and perseveration errors on the Continuous Performance Task. On the other hand, Blume et al. [62] compared response inhibition, attention, decision-making, and impulsivity among four groups of individuals, i.e., obesity and food addiction; obesity and binge eating disorder; obesity/food addiction and binge eating disorder; and obesity only. The authors did not find food addiction to be related to altered executive functioning.

Ruddock et al. [63] evaluated the attentional bias using eye tracking while showing pictures of chocolate among individuals with self-perceived food addiction in design that evaluated state factors like hunger or expectancy of reward or having food addiction. The authors found that the expectancy of receiving chocolate as reward was associated with attentional bias, while hunger state or having self-perceived food addiction was not associated with attentional bias toward food related cues. In another eye tracking paradigm, sad mood induction through showing of a video of child passing away with cancer was associated with attentional bias towards unhealthy food among those with food addiction, but such a change did not occur in those without food addiction [64]. This suggests that emotional cues may impel or prime those with food addiction towards specific food types. In another study, Gearhardt et al. [65] studied food-related visual attention and dwell time of food stuff among obese and overweight women. The authors reported that hunger was associated with attentional bias toward sweets, and trend level attentional bias towards fried (fatty) foods. On the other hand, hunger was associated with shorter dwell time on fried food. Taken together, literature suggests that hunger may be an important component that may influence attentional biases in individuals with food addiction. We acknowledge that though addiction has gained traction, fat addiction is an emerging concept, and nevertheless needs debate and discussion to inform lifestyle practices and research directions.

### 3.2. Craving and Liking

Craving and liking are related, but represent distinct terms that are linked to food addiction. While craving refers to desire or urge to eat a food item, liking refers to qualitative and affective evaluation of food [66,67,68]. Liking for fat has been evaluated in a large web-based study to examine the determinants of dietary patterns and nutritional status [69]. The investigators reported that individuals with a strong liking for fat had high total energy and fat intake, and high consumption of saturated fats, meat, butter, sweetened cream desserts and croissant-like pastries. Such individuals also consumed low quantities of fiber, fruits, vegetables and yogurt. It was highlighted that increased liking for fat, especially fat-and-salt liking, was associated with a lower intake of fruit and vegetables.

Gearhardt et al. [70] assessed craving for 180 food items among a sample of 105 obese or overweight women. The authors found that those with greater symptomatology of food addiction had higher craving ratings for fatty foods. However, as BMI increased, the craving decreased. In contrast to craving in this study, high fat content was not associated with high liking for food product, suggesting a dichotomy between craving and liking.

## 4. Understanding the Physiological and Neurobiological Processes of Fat Food Addiction

**Figure 1:**
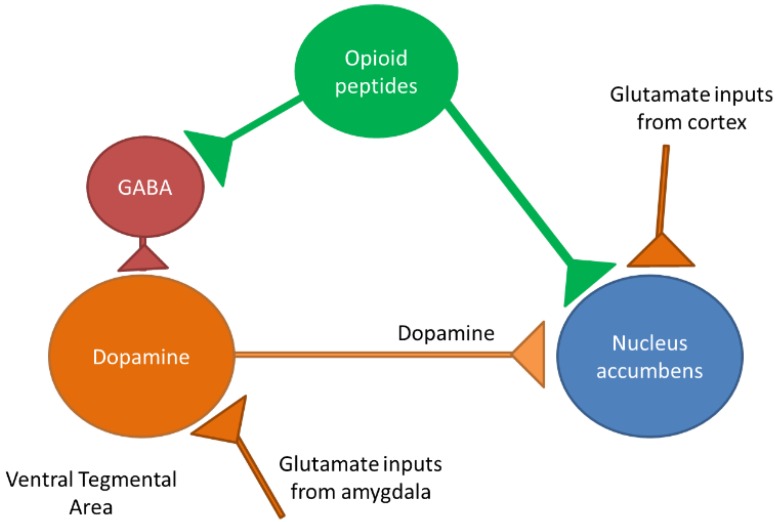
Schematic representation of the reward pathway. The figure shows the interplay between different neurons where the nucleus accumbens seems to be the central player, receiving the projection of dopaminergic, glutamatergic and opioidergic neurons. The model for food addiction might be quite different and is under examination [74].

There have been considerable advances in understanding the mechanisms of addiction for food rich in lipids. Some of them were conducted on animals, particularly rodent models. Other directions of research, for example, genetics and neuroimaging have explored the origin of addiction towards fat and other palatable foods in human participants [71,72]. The reward pathway (schematically shown in Figure 1) is intricately linked to understanding the addiction to fatty food, though some differences have been reported in food addiction and substance use disorders [73].

### 4.1. Animal Models for Understanding the Addiction to Fat Rich Foods

The advantage of animal models is that they are able to develop addiction to fat as the diets given in animal models are homogenous [75,76,77]. This is not possible in human studies. The high-fat diet that generally comprises of 45% of energy from lipids is used to trigger obesity in rodents [78]. However, none of the experimental high-fat diets resembles closely to human diet fatty acid composition, though they are efficient to induce obesity.

Initially, Avena et al. [79] developed a model for sugar addiction which showed patterns of binge eating, withdrawal symptoms, and neurochemical changes similar to those observed with opiate addiction. The phenotype of animal was created by restricting the frequency, duration or access to sugar. Subsequently, fat models of bingeing have been developed in conjunction with carbohydrates, wherein corn oil is used as a reinforcing food item. However, the features of opiate-like withdrawal were not noted by Avena et al. [80] when animals were deprived of food after fat bingeing. This observation suggests that fat addiction may have different phenomenological aspects than the addiction to sugars. An alternate explanation could be that fat addiction might be more closely aligned to behavioral addiction like gambling disorder, while addiction to sugar rich food might be more closely aligned to substance use disorders. The development of animal models has the potential to advance the field substantially, by enabling to better understand the neurobiological alterations, and to assess the changes in bingeing behaviors with medications or other interventions [81]. Yet, one needs to be cognizant of the fact that translation of human behavior of food consumption is much more complex than animals, and is influenced by socio-economic and political environment, and the determinants like cost, availability and marketing. Furthermore, it is possible that modeling addiction in animals (especially rodents) might differ substantially from clinical situation [8]. It has been argued that simplistic experiments would need critical reflection about translational validity of patterns of eating behavior and food choice from animals to humans.

### 4.2. Neurotransmitters Including Dopamine

Animal studies have suggested implication of dopamine in the nucleus accumbens in the rat model of addictive behaviors towards fat [82]. Hence, microdialysis samples were taken in this model before, during and after sham feeding with corn oil. The study found an increase in dopamine in the sham licking group leading to the inference that corn oil increases dopamine concentrations in the nucleus accumbens in a manner similar to those induced by sucrose. In another study, low concentration of non-esterified fatty acid (linoleic acid) increased the dopamine levels in the nucleus accumbens and amygdala in a manner equivalent to those resulting from corn oil in the brain’s reward system [83].

Dela Cruz and colleagues studied the expression of c-Fos in reward circuit areas in rats which were exposed to sugars and fats [84,85]. The authors reported c-Fos like immunoreactivity after consumption of corn oil solutions, isocaloric glucose and fructose, in the dopaminergic mesotelencephalic nuclei (ventral tegmental area) and projections (infralimbic and prelimbic medial prefrontal cortex, basolateral and central-cortico-medial amygdala, core of nucleus accumbens as well as the dorsal striatum), but not in the nucleus accumbens shell. This signified transcriptional activation of the dopaminergic pathway with exposure to certain nutrients including fat.

Dela Cruz et al. aimed at investigating whether dopamine antagonists (D1 receptor antagonist SCH23390 and D2 receptor antagonist raclopride) attenuated the development of fat conditioned flavour preference among rats [86]. These investigators reported that, as compared to sucrose, the D1 and D2 receptor antagonists were not able to attenuate the fat conditioned flavor preference. They further suggested that fat addiction in rats could possibly have distinct mechanisms than sugars, which involved the post ingestive phase.

The role of opioid receptors and fatty food addiction has also been explored [76]. It has been seen that after injection of morphine, a mu-opioid receptor agonist, rats preferred fats over carbohydrates when both were available. Intra-accumbens administration of opioid agonists increased the consumption of fats, and the effect was blocked by the administration of naltrexone, an opioid antagonist [87]. The opioid receptors have been implicated in not only the ‘liking’ process, but also the ‘wanting’ process of excessive food consumption, and the effects are blocked by opioid antagonists.

Endocannabinoid system is another neurotransmitter system studied in relation with animal model of excessive fat consumptive behavior. Ward et al. [88] studied male, cannabinoid (CB1) knockout mice which were trained to respond to the sweet reinforcer (Ensure) or corn oil. The authors suggest that CB1 receptor antagonism selectively attenuated reinstatement of responding for Ensure. Interestingly, the genetic deletion of the CB1 receptor did not attenuate reinstatement of corn-oil seeking. The authors suggest that either CB1 receptor system does not play an equivalent role in modulating conditioned seeking or corn oil may serve as a robust reinforcer. Additionally, Brissard et al. [89] found that invalidation of CB1R gene was related to lower levels of fat preference among mice, and similar results were obtained after using rimonabant, a cannabinoid receptor antagonist. The authors reported that fat taste perception was mediated through calcium signaling and GLP-1 secretion in lingual taste bud cells. Peterschmitt et al. [90] looked at the link between the gustatory and the reward pathway with regard to fat intake. The authors observed that lipid taste perception was based upon the systematic activation of the major cerebral structures of the canonical gustatory pathway and was intricately linked to the reward pathway through the ventral tegmental area.

### 4.3. Neuroimaging Correlates

Though literature exists on the neuroimaging correlates of obesity [91,92], studies on the neuroimaging of food addiction have gradually started to come up. Gearhardt et al. [93] assessed the blood oxygen level-dependent functional magnetic resonance imaging (fMRI) activation in response to receipt and anticipated receipt of palatable food (chocolate milkshake) among adolescent female participants. The investigators demonstrated that food addiction scores correlated with greater activation in the anterior cingulate cortex, medial orbitofrontal cortex and amygdala, consequent to anticipated receipt of food. The participants with high food addiction scores had enhanced activation of dorsolateral prefrontal cortex and caudate, but less activation in lateral orbitofrontal cortex in response to anticipated receipt of food. These findings underscore the similarity of food addiction to other types of addictions, especially in relation to involvement of the reward pathway.

Hsu et al. [94] assessed response inhibition and error processing among subjects with obesity and sweet food addiction by fMRI. Women with obesity and food addiction had a higher score for impulsivity and lower brain activation (processing response inhibition over the right rolandic operculum and thalamus) than controls. The activation during error processing over the left insula, precuneus, and bilateral putamen were higher in the subjects with obesity and sweet food addiction than controls. These findings suggest that women with obesity and sweet food addiction have impaired rolandic operculum activation.

A further study looked at the relationship of food addiction and functional connectivity in the brain during fasting and fed state [95]. The authors found that high number of symptoms of food addiction were associated with ventral caudate-hippocampus hyperconnectivity in the fasted scan only. However, a significant reduction of this connectivity was observed in the fed scans, suggesting that heightened connectivity in the ventral striatum during a fasted state corroborated reward prediction signals, further lending credence to the involvement of the reward pathway.

**Figure 2:**
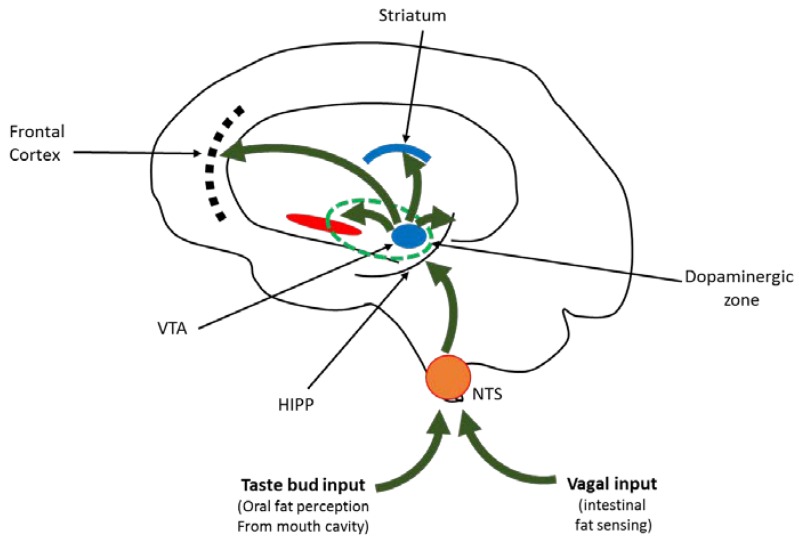
Relationship of food intake and reward pathway. The figure shows that the gustatory memory for fat and its implication would depend on the cues coming from taste bud cells, localized in the lingual papillae, and vagal nerve information from intestinal lipid sensing. Both kinds of information will ascend to different parts of the brain via NTS. Hippocampus will be involved in the learning of palatability of fat, and communicate to VTA which is sending its afferences to frontal cortex, straitum and other parts of the brain. Indeed, the dopaminergic zone covers VTA and NA. NTS: nucleus tractus solitaris; HIPP: hippocampus; VTA: ventral tegmental area.

A schematic representation of the neurobiological relationship of fatty food intake, mediated through gustatory signaling and reward pathway, is presented in Figure 2.

### 4.4. Genetics Underpinnings

Several studies have also looked at the genetic associations of food addiction. A study evaluated whether a composite index of elevated dopamine signaling, a multilocus genetic profile score (MLGP) could segregate between those with food addiction and normal eating behavior [96]. The authors observed that MLGP score was high in subjects with food addiction, and it correlated positively with binge eating, food cravings, and emotional overeating. This finding supported the view that dopamine signaling genetic profile was different in subjects with food addiction.

Pedram et al. [97] studied food addiction in the Newfoundland population and observed the major allele A of rs2511521 located in DRD2 and the minor allele T of rs625413 located in TIR domain containing adaptor protein (TIRAP) to be significantly associated with food addiction. A study on the Asian American college students assessed the relationship of food addiction and a dopamine-resistant receptor (DRD2) polymorphism [98]. The authors reported that DRD2 A1 allele among Asian Americans (versus A2 allele) was associated with greater carbohydrate craving, but not fat craving. Cornelis et al. [99] presented genome wide analysis of food addiction in more than 9000 women with European ancestry. This study showed two loci significant at genome-wide level (17q21.31 and 11q13.4), but they did not have any obvious roles in eating behavior. The study did not find any candidate single nucleotide polymorphism or gene for drug addiction to be significantly associated with food addiction after correction for multiple testing.

There is accruing literature that suggests that reduced fat taste perception may contribute to increased fat consumption and, consequently, to obesity [100], and this might be influenced by the genetic polymorphisms. Studies from USA, Algeria and Tunisia seem to suggest that rs1761667-AA genotype of CD36 receptor is associated with obesity, and high thresholds for oro-sensory detection of dietary lipids [101,102,103]. Interestingly, Plesnik et al. [104] reported that another variant of CD36, i.e., rs1527483 SNP, was associated with greater body weight in young Czech participants. Thus, the taste threshold and preference for fat, mediated through specific genetic polymorphisms, may determine fat-eating behaviors that may lead to fat addiction.

## 5. Conclusions, Limitations and Future Directions

**Figure 3:**
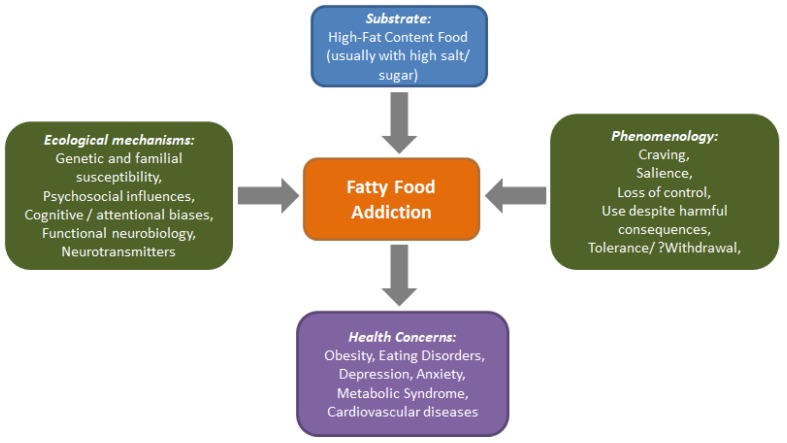
Schematic representation of addiction to fat.

Addiction to food products, especially those rich in fat has received attention in recent decades. Figure 3 depicts the overall associations and implications of addiction to fat replete diets. The construct of food addiction has undergone sufficient scrutiny, and means and measures have been developed to reliably assess this condition. Fat as a component of food addiction itself has yet to find its niche, but has possible implications for the control and prevention of obesity. Research has elaborated on the attentional biases and cognitive functioning in individuals with food addiction, and has pitched varied findings. Animal models of food addiction, especially those which have used fat as a substrate, have expanded the scope of the field and have given an armamentarium of options for understanding the condition and interventional choices. Neuroimaging and genetic studies have also progressed, enriching the field.

Some of the limitations of the present paper should be born in mind while considering different observations. The present findings have not been synthesized as a systematic review, but are rather in the form of a narrative review. The advantage of narrative review is that broad range of findings can be presented to provide the reader with various dimension of the topic, but it may not be able to present all relevant literature in the field. The concept of food addiction, similar or different from other (substance/drug) addiction, as a continuum of behavioral addiction has been debated. Additionally, segregation of food addiction into a specific macronutrient based fat addiction may be difficult to operationalize clinically, as food products generally contain multiple elements together (fats, sugars and salt). Furthermore, food addiction as a concept has been criticized as pathologizing a normal behavior, and some researchers have questioned the validity and nomenclature of the construct [8].

Future research investigations are required to look at the stability of addiction to fat rich food over longitudinal course. The etiological understanding would be strengthened by foray into multi-modal assessment incorporating neuroimaging, genetic and psychological domains. Another aspect would be determining a threshold for fat composition in the food to qualify for fat addiction. Neurobiological studies would be strengthened if they incorporate the neuroimaging responses to palatable food taste, and cues (including visual cues). Neurobiological correlates of fat addiction and its persistence can be elicited by developing studies for individual nutrient components as well as combinations in sweet and savory food and linking it to markers of obesity. It would be pertinent to see how other neuropsychological functions like motivation, sensory processing in various domains and working memory interact with reward and homeostatic systems in controlling the various phenomenological aspects of fat addiction. Also, relationship of addictive behavior with intervention outcomes (for example, for obesity) needs to be looked into. The social impact of food addiction from a macro policy level, and the lived experience of the individual with ‘food addiction’ would help better understand the condition. Also, attempts to enhance the awareness of this condition and the harmful impact of trans-fat and saturated fat, coupled with greater funding to understand and address this issue would help both for primary and secondary prevention. Of course, the overarching aim would be to provide with relevant prevention for at-risk population, and suitable interventions for affected individuals.

## References

[1] Popkin B.M., Adair L.S., Ng S.W. (2012). Global nutrition transition and the pandemic of obesity in developing countries. Nutr. Rev..

[2] Kochhar K.P. (2008). Dietary spices in health and diseases: I. Ind. J. Physiol. Pharmacol..

[3] Kochhar K.P. (2008). Dietary spices in health and diseases (II). Ind. J. Physiol. Pharmacol..

[4] Erlanson-Albertsson C. (2010). Fat-rich food palatability and appetite regulation. Fat Detection: Taste, Texture, and Post Ingestive Effects.

[5] Drewnowski A. (2009). Obesity, diets, and social inequalities. Nutr. Rev..

[6] Popkin B.M. (2006). Global nutrition dynamics: The world is shifting rapidly toward a diet linked with noncommunicable diseases. Am. J. Clin. Nutr..

[7] Popkin B.M., Gordon-Larsen P. (2004). The nutrition transition: Worldwide obesity dynamics and their determinants. Int. J. Obes..

[8] Hebebrand J., Albayrak Ö., Adan R., Antel J., Dieguez C., de Jong J., Leng G., Menzies J., Mercer J.G., Murphy M. (2014). “Eating addiction”, rather than “food addiction”, better captures addictive-like eating behavior. Neurosci. Biobehav. Rev..

[9] Gordon E.L., Ariel-Donges A.H., Bauman V., Merlo L.J. (2018). What Is the Evidence for “Food Addiction?” A Systematic Review. Nutrients.

[10] Lerma-Cabrera J.M., Carvajal F., Lopez-Legarrea P. (2015). Food addiction as a new piece of the obesity framework. Nutr. J..

[11] Bassareo V., Gambarana C. (2019). Food and Its Effect on the Brain: From Physiological to Compulsive Consumption. Front. Psychiatry.

[12] Fernandez-Aranda F., Karwautz A., Treasure J. (2018). Food addiction: A transdiagnostic construct of increasing interest. Eur. Eat. Disord. Rev. J. Eat. Disord. Assoc..

[13] Pelchat M.L. (2009). Food addiction in humans. J. Nutr..

[14] Shriner R., Gold M. (2014). Food addiction: An evolving nonlinear science. Nutrients.

[15] Golay A., Bobbioni E. (1997). The role of dietary fat in obesity. Int. J. Obes. Relat. Metab. Disord. J. Int. Assoc. Study Obes..

[16] Smilowitz J.T., German J.B., Zivkovic A.M., Montmayeur J.-P., le Coutre J. (2010). Food Intake and Obesity: The Case of Fat. Fat Detection: Taste, Texture, and Post Ingestive Effects.

[17] Meule A. (2019). A Critical Examination of the Practical Implications Derived from the Food Addiction Concept. Curr. Obes. Rep..

[18] Cassin S.E., Buchman D.Z., Leung S.E., Kantarovich K., Hawa A., Carter A., Sockalingam S. (2019). Ethical, Stigma, and Policy Implications of Food Addiction: A Scoping Review. Nutrients.

[19] Khan N.A., Besnard P. (2009). Oro-sensory perception of dietary lipids: New insights into the fat taste transduction. Biochim. Biophys. Acta.

[20] Mattes R.D. (2005). Fat taste and lipid metabolism in humans. Physiol. Behav..

[21] Drewnowski A., Mennella J.A., Johnson S.L., Bellisle F. (2012). Sweetness and food preference. J. Nutr..

[22] Sellayah D., Cagampang F.R., Cox R.D. (2014). On the evolutionary origins of obesity: A new hypothesis. Endocrinology.

[23] Reddon H., Patel Y., Turcotte M., Pigeyre M., Meyre D. (2018). Revisiting the evolutionary origins of obesity: Lazy versus peppy-thrifty genotype hypothesis. Obes. Rev. Off. J. Int. Assoc. Study Obes..

[24] Genné-Bacon E.A. (2014). Thinking evolutionarily about obesity. Yale J. Biol. Med..

[25] Clifton P.M., Keogh J.B. (2017). A systematic review of the effect of dietary saturated and polyunsaturated fat on heart disease. Nutr. Metab. Cardiovasc. Dis. NMCD.

[26] Power R., Prado-Cabrero A., Mulcahy R., Howard A., Nolan J.M. (2019). The Role of Nutrition for the Aging Population: Implications for Cognition and Alzheimer’s Disease. Annu. Rev. Food Sci. Technol..

[27] Besnard P., Passilly-Degrace P., Khan N.A. (2016). Taste of Fat: A Sixth Taste Modality?. Physiol. Rev..

[28] Val-Laillet D., Aarts E., Weber B., Ferrari M., Quaresima V., Stoeckel L.E., Alonso-Alonso M., Audette M., Malbert C.H., Stice E. (2015). Neuroimaging and neuromodulation approaches to study eating behavior and prevent and treat eating disorders and obesity. NeuroImage Clin..

[29] Schmidt U., Campbell I.C. (2013). Treatment of eating disorders can not remain “brainless”: The case for brain-directed treatments. Eur. Eat. Disord. Rev. J. Eat. Disord. Assoc..

[30] Rogers P.J. (2017). Food and drug addictions: Similarities and differences. Pharmacol. Biochem. Behav..

[31] Hone-Blanchet A., Fecteau S. (2014). Overlap of food addiction and substance use disorders definitions: Analysis of animal and human studies. Neuropharmacology.

[32] Meule A., Gearhardt A.N. (2014). Food addiction in the light of DSM-5. Nutrients.

[33] Schreiber L.R.N., Odlaug B.L., Grant J.E. (2013). The overlap between binge eating disorder and substance use disorders: Diagnosis and neurobiology. J. Behav. Addict..

[34] Citrome L. (2015). A primer on binge eating disorder diagnosis and management. CNS Spectr..

[35] Carter J.C., Van Wijk M., Rowsell M. (2019). Symptoms of “food addiction” in binge eating disorder using the Yale Food Addiction Scale version 2.0. Appetite.

[36] Linardon J., Messer M. (2019). Assessment of food addiction using the Yale Food Addiction Scale 2.0 in individuals with binge-eating disorder symptomatology: Factor structure, psychometric properties, and clinical significance. Psychiatry Res..

[37] Burrows T., Skinner J., McKenna R., Rollo M. (2017). Food Addiction, Binge Eating Disorder, and Obesity: Is There a Relationship?. Behav. Sci. Basel Switz..

[38] Pursey K.M., Stanwell P., Gearhardt A.N., Collins C.E., Burrows T.L. (2014). The prevalence of food addiction as assessed by the Yale Food Addiction Scale: A systematic review. Nutrients.

[39] Penzenstadler L., Soares C., Karila L., Khazaal Y. (2018). Systematic Review of Food Addiction as Measured With the Yale Food Addiction Scale: Implications for the Food Addiction Construct. Curr. Neuropharmacol..

[40] Zhao Z., Ma Y., Han Y., Liu Y., Yang K., Zhen S., Wen D. (2018). Psychosocial Correlates of Food Addiction and Its Association with Quality of Life in a Non-Clinical Adolescent Sample. Nutrients.

[41] Ayaz A., Nergiz-Unal R., Dedebayraktar D., Akyol A., Pekcan A.G., Besler H.T., Buyuktuncer Z. (2018). How does food addiction influence dietary intake profile?. PLoS ONE.

[42] Pursey K.M., Collins C.E., Stanwell P., Burrows T.L. (2015). Foods and dietary profiles associated with “food addiction” in young adults. Addict. Behav. Rep..

[43] Ivezaj V., Wiedemann A.A., Grilo C.M. (2017). Food addiction and bariatric surgery: A systematic review of the literature. Obes. Rev. Off. J. Int. Assoc. Study Obes..

[44] Pepino M.Y., Stein R.I., Eagon J.C., Klein S. (2014). Bariatric surgery-induced weight loss causes remission of food addiction in extreme obesity. Obes. Silver Spring Md..

[45] Sevinçer G.M., Konuk N., Bozkurt S., Coşkun H. (2016). Food addiction and the outcome of bariatric surgery at 1-year: Prospective observational study. Psychiatry Res..

[46] Guerrero Pérez F., Sánchez-González J., Sánchez I., Jiménez-Murcia S., Granero R., Simó-Servat A., Ruiz A., Virgili N., López-Urdiales R., Montserrat-Gil de Bernabe M. (2018). Food addiction and preoperative weight loss achievement in patients seeking bariatric surgery. Eur. Eat. Disord. Rev. J. Eat. Disord. Assoc..

[47] Gearhardt A.N., Corbin W.R., Brownell K.D. (2009). Preliminary validation of the Yale food addiction scale. Appetite.

[48] Gearhardt A.N., Roberto C.A., Seamans M.J., Corbin W.R., Brownell K.D. (2013). Preliminary validation of the Yale Food Addiction Scale for children. Eat. Behav..

[49] Chen G., Tang Z., Guo G., Liu X., Xiao S. (2015). The Chinese version of the Yale Food Addiction Scale: An examination of its validation in a sample of female adolescents. Eat. Behav..

[50] Nantha Y.S., Patah N.A.A., Pillai M.P. (2016). Preliminary validation of the Malay Yale Food Addiction Scale: Factor structure and item analysis in an obese population. Clin. Nutr. ESPEN.

[51] Brunault P., Ballon N., Gaillard P., Réveillère C., Courtois R. (2014). Validation of the French version of the Yale Food Addiction Scale: An examination of its factor structure, reliability, and construct validity in a nonclinical sample. Can. J. Psychiatry.

[52] Gearhardt A.N., Corbin W.R., Brownell K.D. (2016). Development of the Yale Food Addiction Scale Version 2.0. Psychol. Addict. Behav. J. Soc. Psychol. Addict. Behav..

[53] Wardle J., Guthrie C.A., Sanderson S., Rapoport L. (2001). Development of the children’s eating behaviour questionnaire. J. Child Psychol. Psychiatry.

[54] Cepeda-Benito A., Gleaves D.H., Williams T.L., Erath S.A. (2000). The development and validation of the state and trait food-cravings questionnaires. Behav. Ther..

[55] Schlundt D.G., Hargreaves M.K., Buchowski M.S. (2003). The eating behavior patterns questionnaire predicts dietary fat intake in African American women. J. Am. Diet. Assoc..

[56] Cappelleri J.C., Bushmakin A.G., Gerber R.A., Leidy N.K., Sexton C.C., Karlsson J., Lowe M.R. (2009). Evaluating the Power of Food Scale in obese subjects and a general sample of individuals: Development and measurement properties. Int. J. Obes..

[57] Batterink L., Yokum S., Stice E. (2010). Body mass correlates inversely with inhibitory control in response to food among adolescent girls: An fMRI study. NeuroImage.

[58] Goldman R.L., Canterberry M., Borckardt J.J., Madan A., Byrne T.K., George M.S., O’Neil P.M., Hanlon C.A. (2013). Executive control circuitry differentiates degree of success in weight loss following gastric-bypass surgery. Obes. Silver Spring Md..

[59] Rodrigue C., Ouellette A.-S., Lemieux S., Tchernof A., Biertho L., Bégin C. (2018). Executive functioning and psychological symptoms in food addiction: A study among individuals with severe obesity. Eat. Weight Disord. EWD.

[60] Franken I.H.A., Nijs I.M.T., Toes A., van der Veen F.M. (2018). Food addiction is associated with impaired performance monitoring. Biol. Psychol..

[61] Steward T., Mestre-Bach G., Vintró-Alcaraz C., Lozano-Madrid M., Agüera Z., Fernández-Formoso J.A., Granero R., Jiménez-Murcia S., Vilarrasa N., García-Ruiz-de-Gordejuela A. (2018). Food addiction and impaired executive functions in women with obesity. Eur. Eat. Disord. Rev. J. Eat. Disord. Assoc..

[62] Blume M., Schmidt R., Hilbert A. (2018). Executive Functioning in Obesity, Food Addiction, and Binge-Eating Disorder. Nutrients.

[63] Ruddock H.K., Field M., Jones A., Hardman C.A. (2018). State and trait influences on attentional bias to food-cues: The role of hunger, expectancy, and self-perceived food addiction. Appetite.

[64] Frayn M., Sears C.R., von Ranson K.M. (2016). A sad mood increases attention to unhealthy food images in women with food addiction. Appetite.

[65] Gearhardt A.N., Treat T.A., Hollingworth A., Corbin W.R. (2012). The relationship between eating-related individual differences and visual attention to foods high in added fat and sugar. Eat. Behav..

[66] Havermans R.C. (2011). “You Say it’s Liking, I Say it’s Wanting…”. On the difficulty of disentangling food reward in man. Appetite.

[67] Mela D.J. (2001). Why do we like what we like?. J. Sci. Food Agric..

[68] Pelchat M.L. (2002). Of human bondage: Food craving, obsession, compulsion, and addiction. Physiol. Behav..

[69] Méjean C., Deglaire A., Kesse-Guyot E., Hercberg S., Schlich P., Castetbon K. (2014). Association between intake of nutrients and food groups and liking for fat (The Nutrinet-Santé Study). Appetite.

[70] Gearhardt A.N., Rizk M.T., Treat T.A. (2014). The association of food characteristics and individual differences with ratings of craving and liking. Appetite.

[71] Volkow N., Wang G.J., Fowler J.S., Tomasi D., Baler R. (2011). Food and drug reward: Overlapping circuits in human obesity and addiction. Brain Imaging in Behavioral Neuroscience.

[72] Fortuna J.L. (2010). Sweet preference, sugar addiction and the familial history of alcohol dependence: Shared neural pathways and genes. J. Psychoact. Drugs.

[73] Ahmed S.H., Lenoir M., Guillem K. (2013). Neurobiology of addiction versus drug use driven by lack of choice. Curr. Opin. Neurobiol..

[74] Ziauddeen H., Farooqi I.S., Fletcher P.C. (2012). Obesity and the brain: How convincing is the addiction model?. Nat. Rev. Neurosci..

[75] Morgan D., Sizemore G.M. (2011). Animal models of addiction: Fat and sugar. Curr. Pharm. Des..

[76] Novelle M.G., Diéguez C. (2018). Food Addiction and Binge Eating: Lessons Learned from Animal Models. Nutrients.

[77] De Jong J.W., Vanderschuren L.J.M.J., Adan R.A.H. (2012). Towards an animal model of food addiction. Obes. Facts.

[78] Marques C., Meireles M., Norberto S., Leite J., Freitas J., Pestana D., Faria A., Calhau C. (2016). High-fat diet-induced obesity Rat model: A comparison between Wistar and Sprague-Dawley Rat. Adipocyte.

[79] Avena N.M. (2007). Examining the addictive-like properties of binge eating using an animal model of sugar dependence. Exp. Clin. Psychopharmacol..

[80] Avena N.M., Rada P., Hoebel B.G. (2009). Sugar and fat bingeing have notable differences in addictive-like behavior. J. Nutr..

[81] Wong K.J., Wojnicki F.H.W., Corwin R.L.W. (2009). Baclofen, raclopride, and naltrexone differentially affect intake of fat/sucrose mixtures under limited access conditions. Pharmacol. Biochem. Behav..

[82] Liang N.-C., Hajnal A., Norgren R. (2006). Sham feeding corn oil increases accumbens dopamine in the rat. Am. J. Physiol. Regul. Integr. Comp. Physiol..

[83] Adachi S., Endo Y., Mizushige T., Tsuzuki S., Matsumura S., Inoue K., Fushiki T. (2013). Increased levels of extracellular dopamine in the nucleus accumbens and amygdala of rats by ingesting a low concentration of a long-chain Fatty Acid. Biosci. Biotechnol. Biochem..

[84] Dela Cruz J.A.D., Coke T., Bodnar R.J. (2016). Simultaneous Detection of c-Fos Activation from Mesolimbic and Mesocortical Dopamine Reward Sites Following Naive Sugar and Fat Ingestion in Rats. J. Vis. Exp. JoVE.

[85] Dela Cruz J.A.D., Coke T., Karagiorgis T., Sampson C., Icaza-Cukali D., Kest K., Ranaldi R., Bodnar R.J. (2015). c-Fos induction in mesotelencephalic dopamine pathway projection targets and dorsal striatum following oral intake of sugars and fats in rats. Brain Res. Bull..

[86] Dela Cruz J.A.D., Icaza-Cukali D., Tayabali H., Sampson C., Galanopoulos V., Bamshad D., Touzani K., Sclafani A., Bodnar R.J. (2012). Roles of dopamine D1 and D2 receptors in the acquisition and expression of fat-conditioned flavor preferences in rats. Neurobiol. Learn. Mem..

[87] Zhang M., Gosnell B.A., Kelley A.E. (1998). Intake of high-fat food is selectively enhanced by mu opioid receptor stimulation within the nucleus accumbens. J. Pharmacol. Exp. Ther..

[88] Ward S.J., Walker E.A., Dykstra L.A. (2007). Effect of cannabinoid CB1 receptor antagonist SR141716A and CB1 receptor knockout on cue-induced reinstatement of Ensure and corn-oil seeking in mice. Neuropsychopharmacol. Off. Publ. Am. Coll. Neuropsychopharmacol..

[89] Brissard L., Leemput J., Hichami A., Passilly-Degrace P., Maquart G., Demizieux L., Degrace P., Khan N.A. (2018). Orosensory Detection of Dietary Fatty Acids Is Altered in CB₁R^−/−^ Mice. Nutrients.

[90] Peterschmitt Y., Abdoul-Azize S., Murtaza B., Barbier M., Khan A.S., Millot J.-L., Khan N.A. (2018). Fatty Acid Lingual Application Activates Gustatory and Reward Brain Circuits in the Mouse. Nutrients.

[91] Patriarca L., Magerowski G., Alonso-Alonso M. (2017). Functional neuroimaging in obesity. Curr. Opin. Endocrinol. Diabetes Obes..

[92] Brooks S.J., Cedernaes J., Schiöth H.B. (2013). Increased prefrontal and parahippocampal activation with reduced dorsolateral prefrontal and insular cortex activation to food images in obesity: A meta-analysis of fMRI studies. PLoS ONE.

[93] Gearhardt A.N., Yokum S., Orr P.T., Stice E., Corbin W.R., Brownell K.D. (2011). The Neural Correlates of “Food Addiction”. Arch. Gen. Psychiatry.

[94] Hsu J.-S., Wang P.-W., Ko C.-H., Hsieh T.-J., Chen C.-Y., Yen J.-Y. (2017). Altered brain correlates of response inhibition and error processing in females with obesity and sweet food addiction: A functional magnetic imaging study. Obes. Res. Clin. Pract..

[95] Contreras-Rodriguez O., Burrows T., Pursey K.M., Stanwell P., Parkes L., Soriano-Mas C., Verdejo-Garcia A. (2019). Food addiction linked to changes in ventral striatum functional connectivity between fasting and satiety. Appetite.

[96] Davis C., Loxton N.J., Levitan R.D., Kaplan A.S., Carter J.C., Kennedy J.L. (2013). “Food addiction” and its association with a dopaminergic multilocus genetic profile. Physiol. Behav..

[97] Pedram P., Zhai G., Gulliver W., Zhang H., Sun G. (2017). Two novel candidate genes identified in adults from the Newfoundland population with addictive tendencies towards food. Appetite.

[98] Yeh J., Trang A., Henning S.M., Wilhalme H., Carpenter C., Heber D., Li Z. (2016). Food Cravings, Food Addiction, and a Dopamine-Resistant (DRD2 A1) Receptor Polymorphism in Asian American College Students. Asia Pac. J. Clin. Nutr..

[99] Cornelis M.C., Flint A., Field A.E., Kraft P., Han J., Rimm E.B., van Dam R.M. (2016). A genome-wide investigation of food addiction. Obes. Silver Spring Md..

[100] Khan A.S., Murtaza B., Hichami A., Khan N.A. (2019). A cross-talk between fat and bitter taste modalities. Biochimie.

[101] Love-Gregory L., Abumrad N. (2011). CD36 genetics and the metabolic complications of obesity. Curr. Opin. Clin. Nutr. Metab. Care.

[102] Mrizak I., Šerý O., Plesnik J., Arfa A., Fekih M., Bouslema A., Zaouali M., Tabka Z., Khan N.A. (2015). The a allele of cluster of differentiation 36 (CD36) SNP 1761667 associates with decreased lipid taste perception in obese Tunisian women. Br. J. Nutr..

[103] Melis M., Carta G., Pintus S., Pintus P., Piras C.A., Murru E., Manca C., Di Marzo V., Banni S., Tomassini Barbarossa I. (2017). Polymorphism rs1761667 in the CD36 Gene Is Associated to Changes in Fatty Acid Metabolism and Circulating Endocannabinoid Levels Distinctively in Normal Weight and Obese Subjects. Front. Physiol..

[104] Plesník J., Serý O., Khan A., Bielik P., Khan N.A. (2018). The rs1527483, but not rs3212018, CD36 polymorphism associates with linoleic acid detection and obesity in Czech young adults. Br. J. Nutr..

